# The silent epidemic: Tackling the burden of anaemia in Africa

**DOI:** 10.4102/ajlm.v14i1.2788

**Published:** 2025-04-23

**Authors:** Glenda M. Davison

**Affiliations:** 1 South African Medical Research Council/Cape Peninsula University of Technology (SAMRC/CPUT) Cardiometabolic Health Research Unit, Faculty of Health and Wellness, Cape Peninsula University of Technology, Cape Town, South Africa; 2 Department of Biomedical Sciences, Faculty of Health and Wellness, Cape Peninsula University of Technology, Cape Town, South Africa; 3 Division of Haematology, Faculty of Health, University of Cape Town, Cape Town, South Africa

Anaemia is described as a condition in which the level of haemoglobin drops below the normal value for an individual’s age, sex and physical state. It most often develops as a symptom of an underlying disease or condition and leads to decreased oxygen delivery to tissues resulting in weakness and chronic fatigue.^1^

The World Health Organization (WHO) has identified anaemia as a global health problem which has extensive consequences, including poor health, lack of economic productivity, and increased morbidity and mortality. In 2021, it was predicted that the global prevalence of anaemia was 24%, which translates to 1.9 billion affected individuals, with women and children making up the majority.^2^ The causes of anaemia vary significantly by geographical region, sex and age, with developing countries in sub-Saharan Africa and Asia carrying the highest burden. A recent assessment by the WHO reported that in Africa, 106 million women and 103 million children under the age of 5 years suffered from anaemia, with the primary cause being iron deficiency.^3^

Besides nutritional deficiencies including iron, vitamin B12 and folate, the underlying causes of anaemia are complex and include haemoglobinopathies, parasitic infections, viruses, drugs, and non-communicable diseases such as malignancy and chronic renal disease. In women of childbearing age, heavy menstrual loss, post-partum bleeding and pregnancy can cause anaemia and, if not treated, can lead to complications for both the newborn baby and the mother.^3^

In Africa, the causes are highly complex and interrelated. For example, parasitic infections such as malaria and helminths can result in haemolysis and blood loss, leading to iron deficiency. Dual infection with *Plasmodium falciparum* and hookworm (*Ancylostoma duodenale* and *Necator americanus*) is well described and has been identified as a leading cause of anaemia, particularly among school children.^4^ To further complicate the problem, Africa has a high burden of genetic haemoglobin disorders such as thalassemia and sickle cell disease. These inherited anaemias, which predominate in Central, East and West Africa, result in reduced red cell lifespans as well as chronic inflammation and haemolysis.^1^ Although haemoglobinopathies are not prominent in southern Africa, the prevalence of HIV and tuberculosis remains high. HIV is closely associated with tuberculosis and both result in chronic anaemia resulting from abnormal iron metabolism and a suppressed bone marrow.^5^ In addition, although the administration of antiretroviral therapy can reduce anaemia in HIV patients, some classes of antiviral drugs can cause it to occur.^6^

Besides infectious disease, non-communicable disorders such as chronic kidney disease are rising rapidly in Africa.^7^ Chronic kidney disease leads to a reduction in the production of erythropoietin, a hormone essential for red cell production and erythropoiesis in bone marrow. Consequently, chronic kidney disease has been identified as an increasingly important cause of anaemia, particularly in the elderly, and is associated with hypertension, HIV, diabetes and autoimmunity.

Despite the high prevalence of anaemia in Africa, it is often seen as a neglected condition, particularly in young children under 5 years and women. Although by 2030 the WHO aims to reduce anaemia in women and children by half, progress is slow.^8^ Most strategies have focused on improving nutrition with iron fortified foods and, while this is important, iron deficiency is only one cause of anaemia; to effectively tackle the problem, research is needed to examine the interplay between all the different causes of the condition. This is particularly important on the African continent, which has a high burden of infectious, nutritional and non-communicable diseases.

The continent of Africa is diverse, and one barrier to achieving these goals is that regional and population-specific reference ranges for haemoglobin and full blood count parameters are lacking. Red blood cell parameters can vary significantly according to geographic region, sex as well as age and, if not interpreted correctly, could result in an overestimation or underestimation of the problem.^2^ Population-specific reference ranges must therefore be developed to remove uncertainty and create a true and relevant definition of anaemia. Recently, the WHO published a guideline for haemoglobin cutoffs; however further clarity, particularly in Africa, is required.^9^ African scientists have a vital role to play in this task.

The causes of anaemia are multifactorial, and early detection can lead to prompt treatment, prevention of severe anaemia, and the timely identification of an underlying disorder which requires further investigation. It has therefore been proposed that haemoglobin screening should be included in all antenatal clinic visits for women. This can be difficult in rural areas where there is no access to a laboratory and, therefore, the incorporation of point-of-care haemoglobin screening may provide a solution. In Kenya, point-of-care haemoglobin testing was incorporated into a panel of tests which included syphilis, HIV and malaria. This was offered to all pregnant women attending seven sites in rural Western Kenya. After 8 months, the results demonstrated a significant increase in testing with early identification of anaemia and a significant improvement in clinical management.^10^ It has also been proposed that haemoglobin or haematocrit testing, at the bedside, be performed for all paediatric and emergency hospital patients, with the goal of implementing effective blood management and the prevention of unnecessary blood transfusions which are still an essential treatment for severe acute anaemia.^11^ Haemoglobin screening using point of care testing has been utilised in several African regions; however, an analysis of these reports indicated that the haemoglobin values were often inaccurate and clinically unusable. The major challenges were errors in sampling, inadequate training and the stability of reagents.^12^ These obstacles must be addressed before point-of-care anaemia screening can be used effectively. One solution is to offer focused and follow-up training for the healthcare professionals performing these tests.

Anaemia remains a global health challenge, particularly in low- and middle-income countries. To support the United Nations in achieving its Sustainable Development Goals,^13^ particularly Goal 2 (Zero Hunger) and Goal 3 (Good Health and Well-being) by 2030, urgent attention must be given to the complex and multifactorial nature of anaemia. African scientists and healthcare professionals are essential in tackling this burden, and the building of strong partnerships among African and regional organisations is critical for effective progress.
